# Impact of Plastic Surgery of Nail Folds Combined with Conservative Nail Plate Reconstruction on the Quality of Life in Patients with Ingrown Toenails: A DLQI-Based Study

**DOI:** 10.3390/jcm14248916

**Published:** 2025-12-17

**Authors:** Tomasz Trochanowski, Aleksandra Dańczak-Pazdrowska, Ewa Baum

**Affiliations:** 1 Poznan University of Medical Sciences, Department of Social Sciences and the Humanities, 60-806 Poznan, Poland; ebaum@ump.edu.pl; 2 Zrodlana Medical Centre, 65-734 Zielona Gora, Poland; 3 Top Medical Clinic, London TW7 5AG, UK; 4 Poznan University of Medical Sciences, Department of Dermatology, 60-355 Poznan, Poland; aleksandra.danczak-pazdrowska@ump.edu.pl

**Keywords:** ingrown toenail, nail fold plastic surgery, conservative nail plate reconstruction, quality of life, DLQI, aesthetic podiatry

## Abstract

**Background/Objectives**: Ingrown toenails are a common podiatric condition that can significantly impair the quality of life of affected patients. The aim of the study was to assess the impact of plastic surgery of the nail folds, preceded by conservative nail plate reconstruction, on the quality of life of patients with ingrown toenails. **Methods**: The DLQI was assessed before (*n* = 108) and after treatment (*n* = 107). The procedure combined plastic surgery of the nail folds with conservative nail plate reconstruction. **Results**: A significant improvement was observed in DLQI scores (*p* < 0.0001) between baseline (DLQI1) and at least 6 months post-procedure (DLQI2). Quality of life improved in all assessed domains, with an average score reduction of 10.09 points. Thirteen patients did not achieve the minimal clinically important difference (MCID). Significant differences in DLQI2 outcomes were also observed by age (*p* = 0.009) and gender (*p* = 0.025). **Conclusions**: Plastic surgery of the nail folds combined with conservative nail plate reconstruction proved effective in improving quality of life in patients with ingrown toenails.

## 1. Introduction

Ingrown toenails are associated with bothersome symptoms, such as pain and inflammation [1,2,3,4,5,6]. These symptoms often lead to changes in patients’ daily activities, affecting both private (e.g., daily functioning) and professional life (e.g., work and school attendance), and may contribute to a decline in overall quality of life [1,2,3,5,7,8,9,10,11,12,13,14,15,16,17]. Pain may also impair gait, as pathological changes are often located in weight-bearing areas of the foot, thereby permanently altering the physiological walking pattern [11,18,19,20,21].

Patients with ingrown toenails typically seek care in podiatric or medical clinics, where treatment options include both conservative measures for milder cases and surgical interventions for more advanced disease [1,5,7,16,22,23,24,25,26,27,28]. Conservative methods include placing wisps of cotton or other material under the ingrown lateral nail edge, putting a brace on the nail, reconstruction of the nail plate, and taping of the nail folds [1,6]. Pharmacological therapy, either systemic or topical, is often initiated to manage bacterial infection of the nail folds [29]. Surgical methods can be divided into two main categories: procedures involving thee nail plate and procedures that preserve the nail plate but correct the nail folds; some techniques combine both approaches by removing parts of the nail plate along with soft tissues [1,3,6,8,9,30,31,32,33]. Treatment methods differ in terms of duration, effectiveness, and risk of possible complications [1]. Complications are usually less frequent after surgical correction of the nail folds than after procedures involving partial or total nail removal [3,10,30].

The aim of this study was to assess the impact of plastic surgery of the nail folds combined with reconstruction of the nail plate on the quality of life of patients with ingrown toenails.

**Figure 1:**
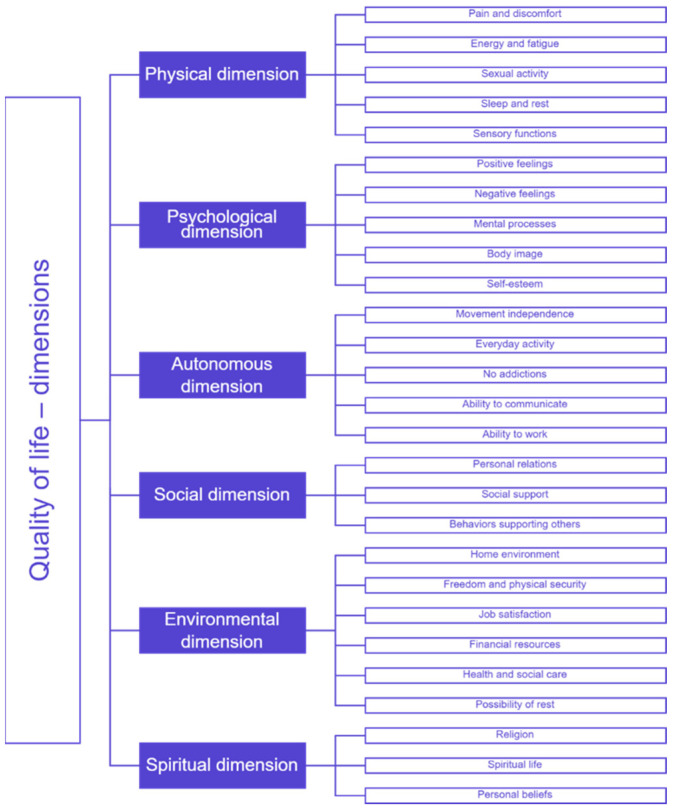
Quality of life—dimensions and categories [34].

Quality of life is a multidimensional concept that may be perceived differently depending on the cultural context in which a patient was raised or currently lives [34]. This study refers primarily to the concept of quality of life as defined in Western culture [34]. However, it should be emphasized that in the current period of globalization and mutual interpenetration of cultures, including the increasing presence of other cultures within Western culture due to strong migration trends, the concept of quality of life is increasingly understood in more universal terms [34]. The impact of ingrown toenails and other foot disorders on quality of life may involve many of its dimensions and categories: physical, psychological, autonomous, social, environmental, and spiritual [34]. Figure 1 illustrates the aforementioned dimensions and division into more detailed categories [34]. However, it is important to note that Figure 1 provides only a general conceptual framework for quality of life, whereas the specific assessment dimensions used in this study are based solely on the DLQI.

## 2. Materials and Methods

The study included 108 patients aged 16–69 years who underwent treatment for ingrown toenails between November 2020 and December 2021. All procedures were performed by a single physician at an outpatient facility (Reference Level Clinic No. 1). The applied treatment method consisted of plastic surgery of the nail folds preceded by conservative nail plate reconstruction.

Quality of life was assessed using the standardized Dermatology Life Quality Index (DLQI), translated into Polish. Patients completed the DLQI questionnaire before the procedure (DLQI1) and again during follow-up (DLQI2). The follow-up assessment was conducted 6–9 months after surgery, with a median follow-up time of 7 months (IQR: 6–8 months). A total of 108 patients completed DLQI1, and 107 completed DLQI2 (one patient did not return the post-treatment questionnaire). The DLQI was administered as part of the routine medical interview and served as an integral component of the patients’ medical documentation.

**Figure 2:**
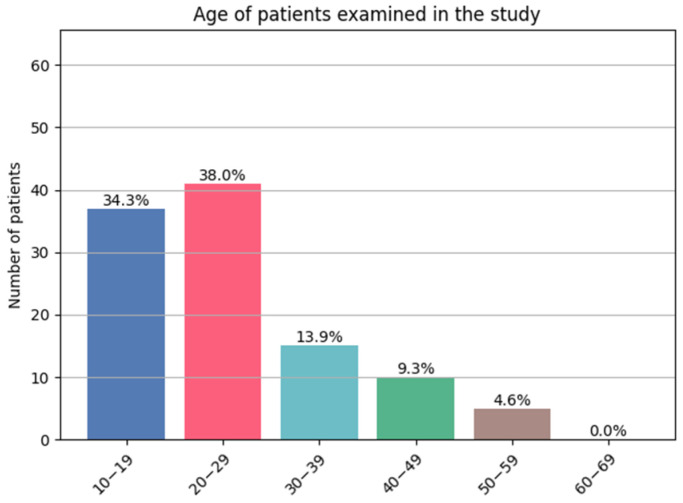
Patient’s age (decades).

In line with DLQI requirements, only patients aged ≥ 16 years were eligible for inclusion. The characteristics of the patients are presented in Table 1 and Figure 2.

The study was approved by the Bioethics Committee of the University of Zielona Gora (no. KB-UZ/4/2021). The following medical equipment was used in the study: Arkada’s Cube (AArkada, Sp.z.o.o., Zielona Gora, Poland). The following software was used in the study: Microsoft Office, version 16.78.3 (Microsoft Corporation, Redmond, WA, USA); Python, version 3.13.4 (Python Software Foundation); and Matplotlib, version 3.10.7 (Matplotlib Development Team).

### 2.1. DLQI Questionnaire

**Figure 3:**
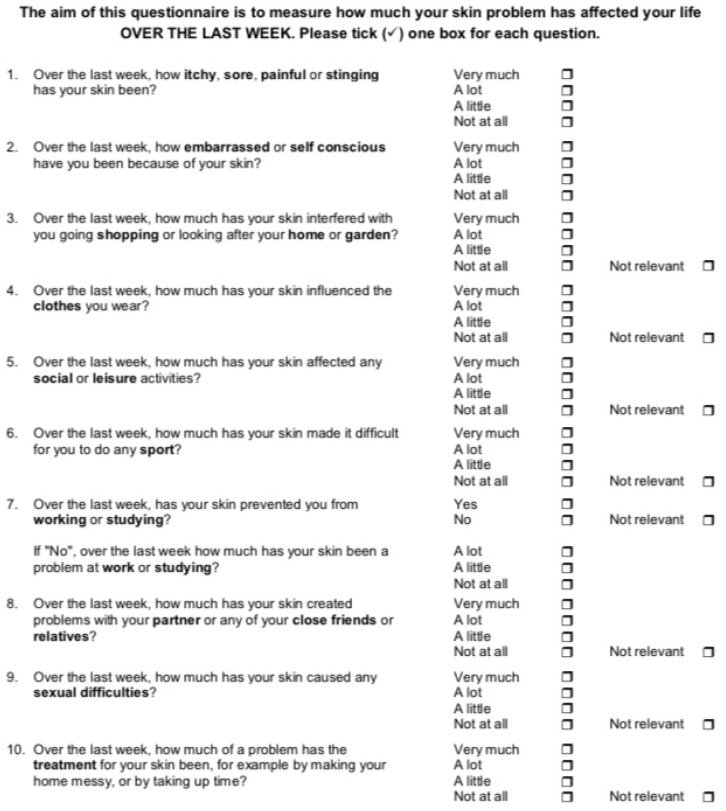
Dermatology Life Quality Index (DLQI) [35].

The standard DLQI consists of 10 questions assessing the quality of life of patients with dermatological diseases [35,36,37,38,39,40,41,42]. The questionnaire evaluates six domains: symptoms and feelings, daily activities, leisure, work and school, personal relationships, and treatment. Each question is scored from 0 to 3, giving a maximum total score of 30, with higher scores indicating greater impairment of quality of life. Interpretation of the results, as proposed by the authors of the DLQI, is shown in Figure 1. According to the DLQI authors, a change of at least 4 points between pre- and post-treatment scores represents the minimal clinically important difference (MCID) and therefore reflects a meaningful improvement in a patient’s quality of life [37,40]. The structure of the DLQI and the interpretation of its scores are presented in Figure 3, Table 2 and Table 3.

### 2.2. Description of Plastic Surgery of the Nail Folds Preceded by Conservative Nail Plate Reconstruction

**Figure 4:**
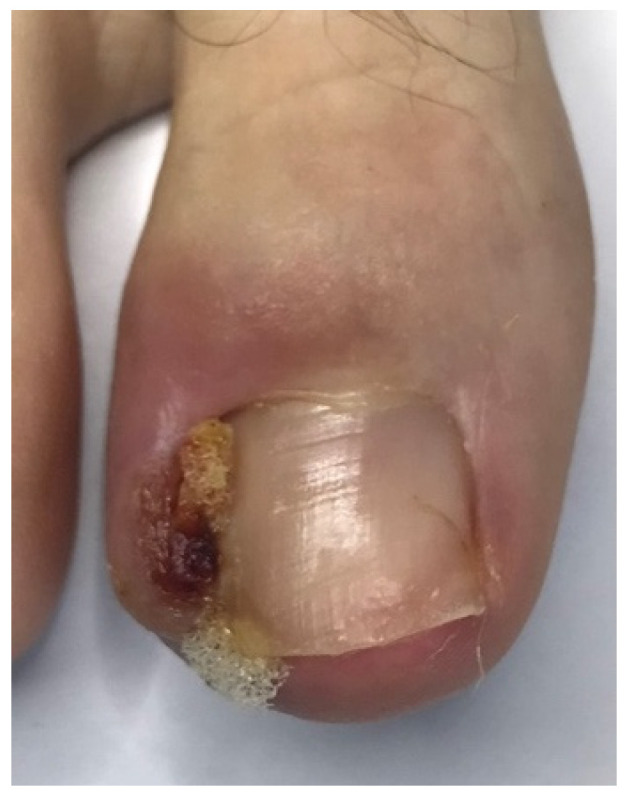
Before the procedure—visible signs of ingrown toenail and inflammation.

**Figure 5:**
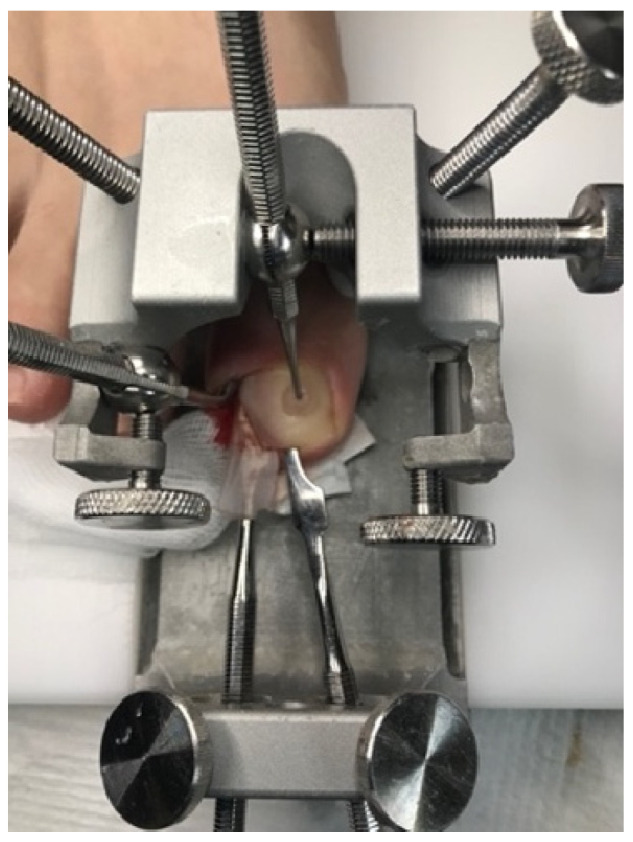
Conservative nail plate reconstruction by Arkada’s method—restoration of the nail plate’s proper shape and filling defects.

**Figure 6:**
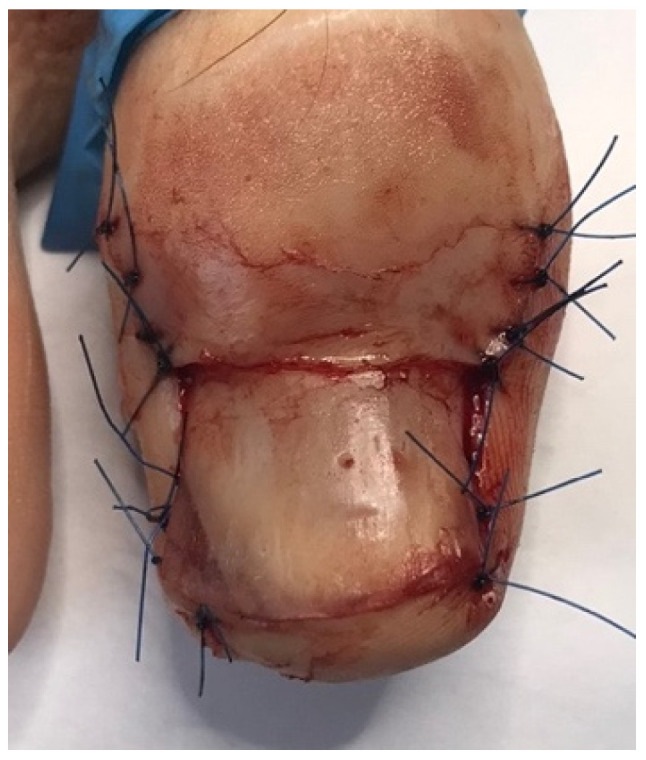
Immediately after plastic surgery of the nail folds.

**Figure 7:**
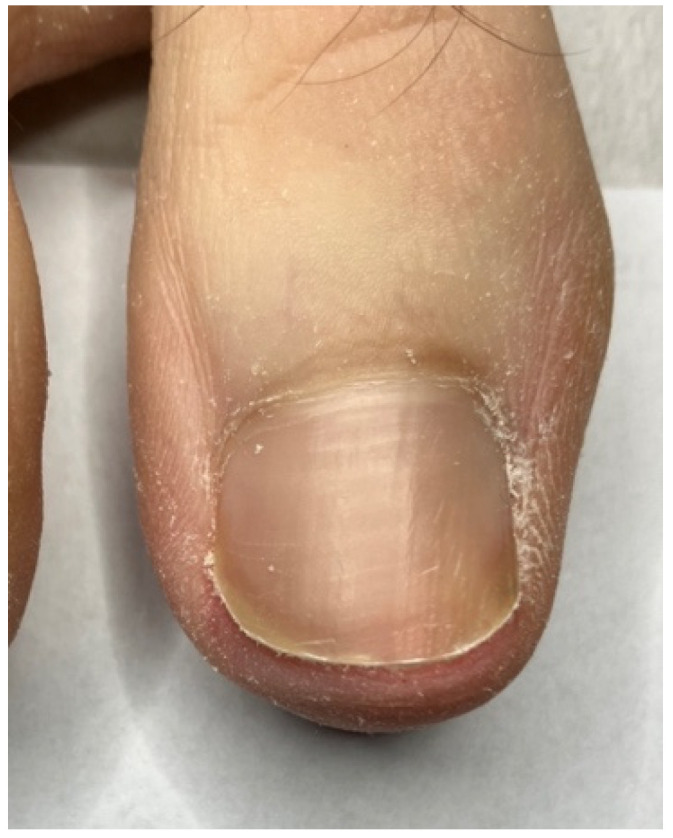
Final appearance six months post-treatment—complete healing and satisfactory aesthetic outcome.

In the studied patients, plastic surgery of the nail folds was always preceded by conservative nail plate reconstruction using Arkada’s method [5,6,43]. This preparatory step offered several advantages. First, it restored the nail plate, which was often altered by inflammation, by filling defects, regaining its natural hardness, and reproducing its original shape. Second, reconstruction facilitated the planning of precise and ergonomic surgical incision lines by re-establishing the correct nail plate shape, thus avoiding both excessive and insufficient excision of the nail folds. Third, the protective layer of composite or acrylic covering the nail plate reduced the risk of intraoperative damage. Finally, this approach promoted faster healing by preventing penetration of damaged nail fragments into the surgical wound. The treatment process is illustrated in Figure 4, Figure 5, Figure 6 and Figure 7.

### 2.3. Statistical Analysis

The normality of the distribution of the studied variables was checked using the Shapiro–Wilk test. Quantitative variables with a lack of normal distribution or on an ordinal scale were presented using the median (min-max) or median (Q25–Q75). Categorical parameters were described as *n* (%). The statistical significance of the studied relationships and differences was checked at the significance level of α = 0.05. In the study for quantitative variables with normality of distributions, parametric tests were used: ANOVA for variables associated with factors and nonparametric tests for quantitative variables with a lack of normality or for variables on an ordinal scale: Mann–Whitney, Wilcoxon and Spearman’s rank correlation coefficient. To examine the interdependence of qualitative characteristics, the chi-square test with Yates’ correction, the chi-square test and the Fisher exact test were used. Dell Inc. (2016) software was used for calculations. All statistical analyses were performed using *Statistica* (version 13; Dell Inc., Round Rock, TX, USA).

## 3. Results

### 3.1. DLQI1

**Figure 8:**
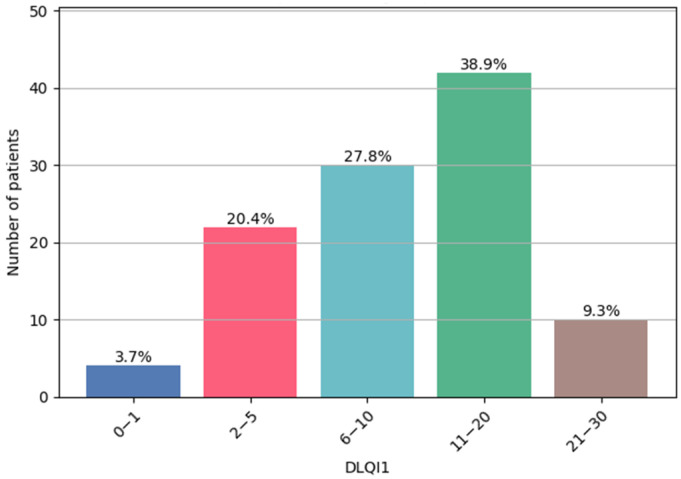
DLQI1—patient subgroup distribution.

Based on DLQI1 scores, patients were classified into five subgroups of quality-of-life impairment. The largest subgroup comprised 42 patients who reported a very high impact (11–20 points). Moderate impact (6–10 points) was reported by 30 patients, while 22 patients experienced a small impact (2–5 points). Only 4 patients reported no impact (0–1 points). At the other extreme, 10 patients experienced an extremely high impact (21–30 points). DLQI1 scores are illustrated in Figure 8.

**Figure 9:**
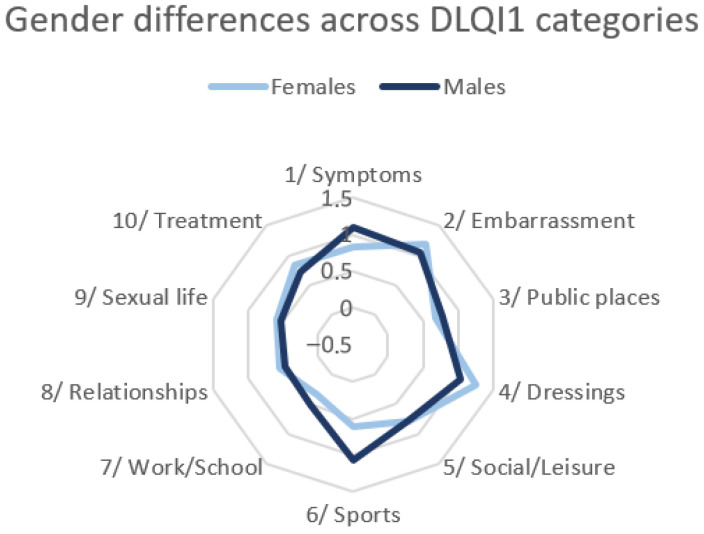
Gender differences in quality of life across DLQI1 categories.

In DLQI1, women more frequently reported embarrassment related to their skin (appearance of the nail folds) and difficulties with appropriate dressing, such as choosing shoes, whereas men more often reported problems with practicing sports. Gender differences in DLQI1 are illustrated in Figure 9.

### 3.2. DLQI2

**Figure 10:**
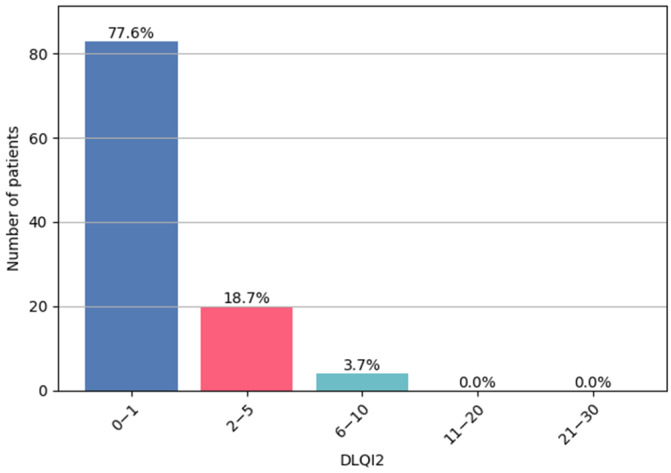
DLQI2—patient subgroup distribution.

The largest subgroup comprised 83 patients whose ingrown toenail symptoms had no impact on their quality of life (0–1 points), followed by 20 patients who reported a small impact (2–5 points). Only 4 patients reported a moderate impact (6–10 points). No patients were classified into the categories of very high (11–20 points) or extremely high (21–30 points) impact. DLQI2 scores are illustrated in Figure 10.

**Figure 11:**
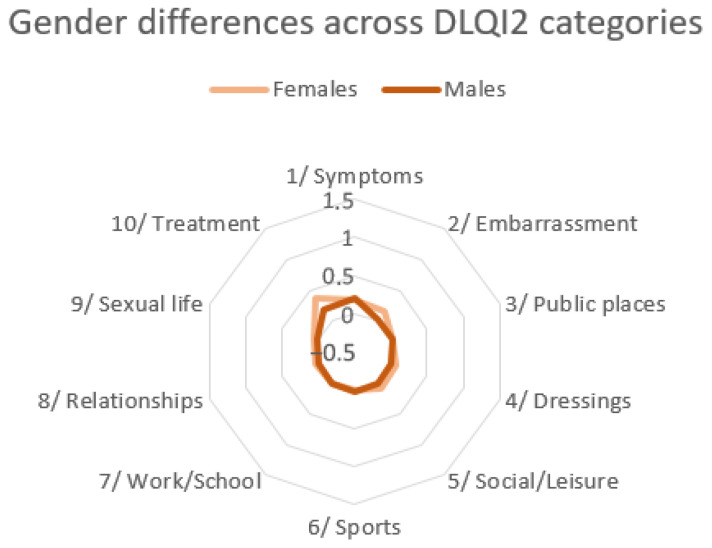
Gender differences in quality of life across DLQI2 categories.

In contrast to DLQI1, the distribution of DLQI2 scores across all categories was similar in both women and men. Gender differences in DLQI2 are illustrated in Figure 11.

### 3.3. Comparison of DLQI1 and DLQI2 Median Values

**Figure 12:**
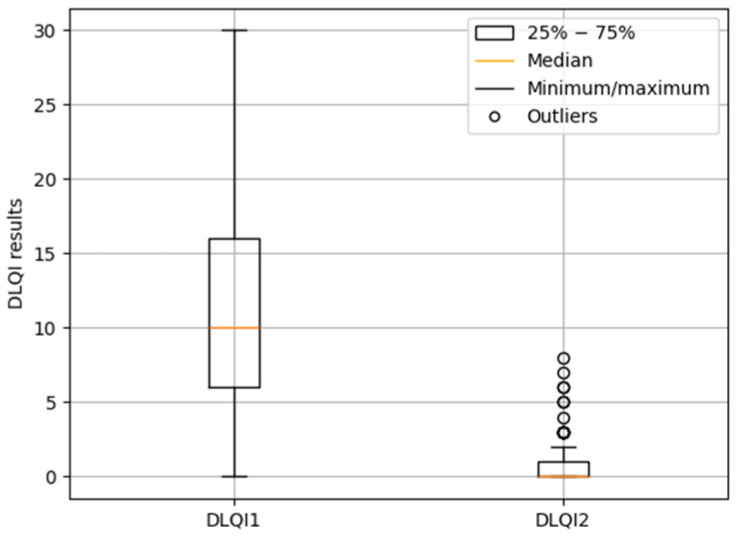
Median DLQI1 and DLQI2 scores.

The Wilcoxon test revealed a significant difference between DLQI1 and DLQI2 scores (*p* < 0.0001). The median DLQI score decreased from 10 before treatment (DLQI1) to 0 after treatment (DLQI2), as illustrated in Figure 12.

**Figure 13:**
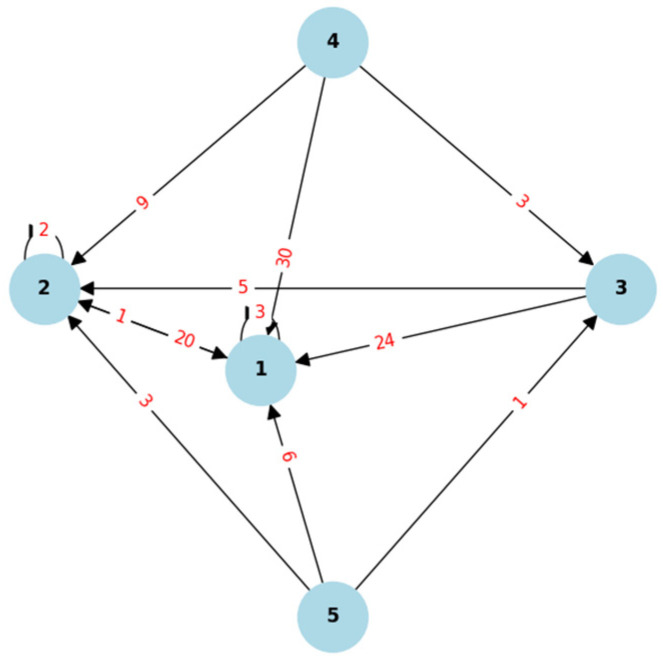
Patient migration between DLQI subgroups before and after treatment.

**Figure 14:**
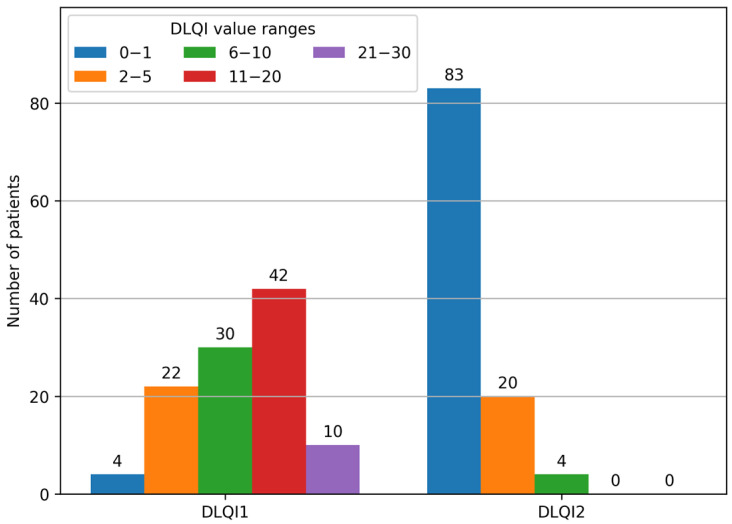
Distribution of patients by DLQI subgroup before and after treatment.

The mean difference in DLQI scores between DLQI1 and DLQI2 across the study population was 10.09 points, with a maximum observed difference of 25 points. Patient migration between DLQI subgroups and final patient distribution by DLQI subgroup before and after treatment are illustrated in Figure 13 and Figure 14, respectively.

In 13 patients, no minimal clinically important difference (MCID) between DLQI1 and DLQI2 was observed. In this subgroup, the mean change was 2.52 points (range: −1.0 to 3.0). It should be noted that these patients already reported relatively good quality of life prior to the treatment. The detailed results are presented in Table 4.

**Figure 15:**
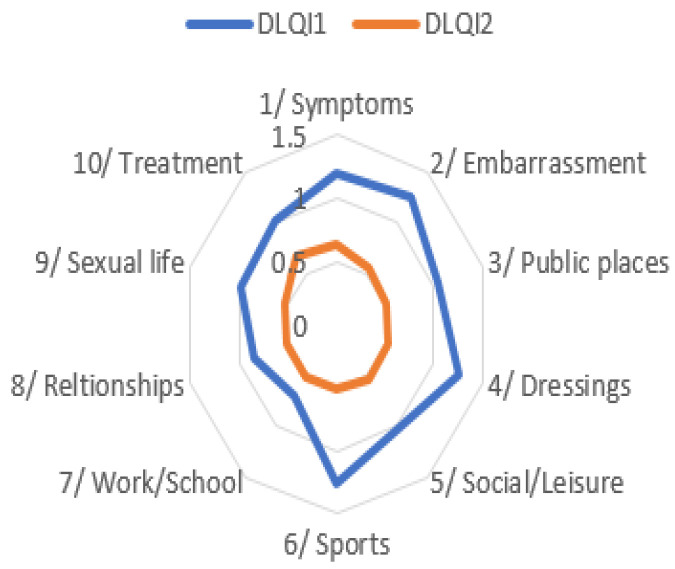
Changes in individual DLQI category scores between DLQI1 and DLQI2.

Comparison of overall DLQI1 and DLQI2 results showed that quality of life improved across all domains. In DLQI1, the greatest impairments were reported in categories related to embarrassment about appearance, difficulties with clothing choice, and limitations in sports activities, whereas the least impact was observed in the domain of work and school performance. In contrast, DLQI2 results demonstrated a more uniform picture across domains, with no single category significantly distinguished. The comparison between DLQI1 and DLQI2 is illustrated in Figure 15.

**Figure 16:**
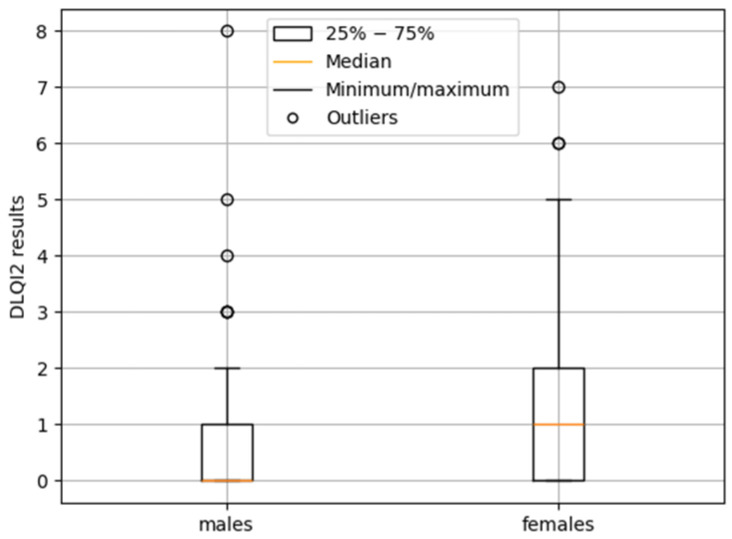
Gender-related differences in median DLQI2 scores.

Statistical analysis of DLQI1 results did not reveal significant differences between women and men (*p* > 0.05). Analysis of DLQI2 results using the Mann–Whitney test confirmed a significant difference between genders (*p* = 0.025). Men were more frequently represented in the 0–1 point range (no impact on quality of life), whereas women were more commonly found in the 2–5 point range (small impact on quality of life). Gender-related differences in median DLQI2 scores are illustrated in Figure 16.

**Figure 17:**
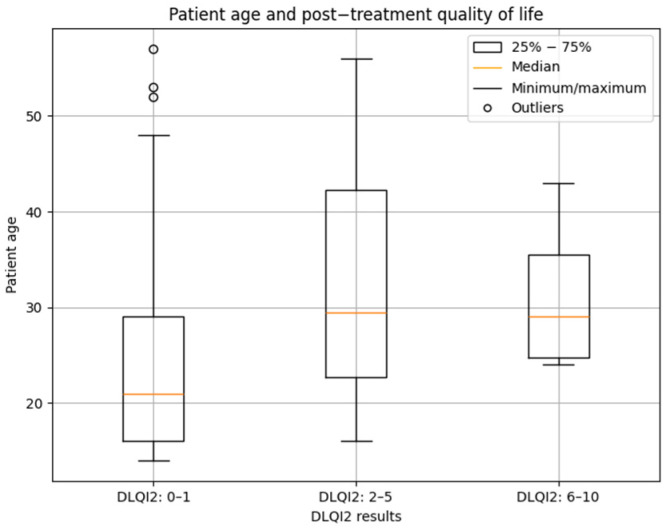
Relationship between patient age and post-treatment quality of life (DLQI2).

An age-based analysis of patients was also performed. The Kruskal–Wallis test did not reveal any significant differences in age between groups in DLQI1 (*p* = 0.0926). In contrast, analysis of DLQI2 results showed a significant difference between groups (*p* = 0.0038). Dunn’s post hoc test confirmed that older patients were more frequently represented in the group with a small impact on quality of life (2–5 points), whereas younger patients predominated in the group with no impact on quality of life (0–1 points) (*p* = 0.009). The median age in the former group was 29.5 years, compared with 21 years in the latter one. Figure 17 illustrates the relationship between patient age and post-treatment quality of life.

## 4. Discussion

The present study evaluated the quality of life of patients suffering from ingrown toenails, with particular attention to gender-related differences. Prior to treatment, women more frequently than men reported complaints related to embarrassment about the appearance of their toenails and the need to change clothing, as reflected in DLQI1 responses. In this context, the need to change clothing primarily referred to footwear selection, especially the choice between open and closed shoes, which can conceal the disease-related appearance of the toes and nails. Since women more often wear open shoes, including in professional settings, this factor may account for the gender differences observed in pre-treatment quality of life.

After treatment, however, these differences diminished. In DLQI2, the distribution of scores across categories was more uniform between men and women, and the earlier gender-specific concerns were no longer as evident. This suggests that surgical correction of the nail folds, preceded by conservative nail plate reconstruction, effectively addressed not only the physical symptoms but also the psychosocial burdens of the condition, contributing to improved quality of life regardless of gender.

Although ingrown toenails can often be concealed from others, giving the impression that they pose little or no burden, their impact on affected individuals is far from negligible. The relatively low visibility of this condition may contribute to its underestimation in broader medical and social contexts, where more attention is directed toward diseases perceived as more serious. However, for patients suffering from ingrown toenails (as well as from other onychopathies), the problem is rarely trivial. It can significantly disrupt daily functioning, reduce overall well-being, and contribute to low mood [1,2,7,10,44]. Moreover, the aesthetic aspect of the feet plays an important role: many patients feel compelled to cover their “unaesthetic” toes due to embarrassment or shame. This highlights that quality of life in patients with ingrown toenails is influenced not only by physical symptoms and functional limitations but also by the cosmetic appearance of the toes, both before and after treatment [3,4,5,6,7,10,11,22,25,38,45,46,47,48].

It should be emphasized that the reduction in quality of life and the unsatisfactory appearance of the toe may result not only from the primary symptoms of ingrown toenails but also from previous treatments, both conservative and surgical, which were often conducted improperly [1]. Such inadequate interventions may exacerbate the initial symptoms, be repeated in an ineffective manner, and ultimately fail to achieve complete recovery [1,22,25,49]. Therefore, when selecting and applying therapeutic strategies (not only for ingrown toenails), it is crucial to consider not only the objective aspect of clinical healing but also the aesthetic outcome. Importantly, these two elements, symptom resolution and cosmetic improvement, do not necessarily improve at the same time [3,5,25,30,33,49,50,51].

Complications of treatment, particularly permanent anatomical changes in the toe and nail following the procedure, are less common after surgical correction of the nail folds than after procedures involving partial or total nail removal [1,3,8,9,10,30,49]. Among the most unsightly and functionally problematic post-procedure complications are permanent narrowing of the nail plate and irregular regrowth of the nail in multiple segments, often resulting from unsuccessful destruction of the nail matrix [25,49,50]. It is therefore not surprising that when chronic symptoms of inflammation and pain persist for months or even years, and treatment proves not only ineffective but also aggravates the initial condition, patients become increasingly fatigued by this prolonged course. Such chronicity significantly limits their ability to perform everyday and professional activities, further worsening their quality of life [1,2,3,4,10,11,25,33,46,52].

By comparing the results of the DLQI completed by patients before and after the procedure, both a statistically significant difference between DLQI1 and DLQI2 and an improvement in quality of life across all assessed domains were observed, thereby confirming the effectiveness of the treatment method applied in this study [5,6].

In addition to the gender differences observed and discussed above in individual categories, other significant differences observed after treatment in the assessment of quality of life using the DLQI also deserve discussion. In particular, the difference in general perception between women and men is noteworthy: on average, men reported a greater improvement in quality of life after the procedure than women. It is worth considering whether this difference reflects men’s generally higher satisfaction with the improvements achieved, or whether it may instead be related to a tendency among men to pay less attention to residual “imperfections” in their health status after treatment. The scientific literature on quality of life (not limited to foot diseases or ingrown toenails) generally suggests that women experience and report a decline in quality of life more frequently and with greater intensity than men, therefore the absence of a full recovery across all domains of quality of life, including an immediate return to the pre-disease state, may be perceived as more burdensome and distressing for women [13,14,45,53,54,55].

Another significant difference observed in the study concerned the age of the patients and can be interpreted as follows: the younger the patient, the greater the likelihood of perceiving an improvement in quality of life after treatment. This relationship, frequently noted in the scientific literature, may be explained by several factors associated with the natural aging process. These include the higher prevalence of comorbidities (both foot-related and systemic), age-related changes in foot biomechanics, structure, and function (which may increase the risk of falls), and slower wound healing; furthermore, younger patients typically heal faster due to better tissue elasticity, fewer concurrent diseases, and greater overall mobility [13,14,16,56,57,58,59,60,61,62].

The present study introduces several innovative elements. Firstly, to the best of our knowledge, no previous research has assessed the quality of life in patients with ingrown toenails treated with the comprehensive method of plastic surgery of the nail folds preceded by conservative nail plate reconstruction [5,6]. Furthermore, this combined therapeutic approach, consisting of both a conservative and a surgical component, is only rarely described in the scientific literature [5,6]. In general, studies addressing the quality of life of patients with ingrown toenails remain limited, and there are relatively few publications specifically evaluating quality of life with standardized questionnaires (Patient-Reported Outcome Measures) in patients undergoing surgical correction of nail folds [2,3,12,53,63,64]. Finally, the aesthetic outcomes of the toe and nail plate, as well as their relationship with ingrown toenail treatment, are not commonly considered in the literature [1,33]. Most studies focus exclusively on clinical recovery, without reference to aesthetic improvement [3,6,9,22,25,33,49,50,51,65,66].

This study also has several limitations. First, only patients qualified for plastic surgery of the nail folds were included. The quality of life of patients with ingrown toenails treated using other methods was not assessed. The study is uncontrolled and observational, and without the above-mentioned comparative studies it is difficult to determine whether the improvement in DLQI scores can be attributed specifically to the combined treatment method presented. Future randomized studies would therefore be valuable to compare the impact of different treatment approaches on patients’ quality of life, particularly considering the basic distinction between surgical methods involving nail intervention (including permanent matrix destruction, e.g., with phenol) and those without nail intervention, in which only the nail folds are corrected, as in the present study.

Second, a broader cross-section of patients with regard to social, professional, and other demographic factors would enhance the findings.

Third, all patients were treated by a single physician in a single outpatient clinic. While this ensured methodological consistency, it also limited the ability to assess variability across different operators or clinical settings. As mentioned above, however, the treatment method employed is rarely used in clinical practice, making a multicenter study difficult to conduct. Nevertheless, future multicenter studies are necessary to validate the generalizability and reproducibility of the treatment method presented.

Another limitation of the study was the use of the DLQI, a questionnaire designed for dermatological conditions in general [35,37,38,39,40,41,42]. Ingrown toenails share many features with dermatological disorders, as the problem involves both the nail plate and the surrounding skin of the nail folds. Although quality-of-life questionnaires dedicated specifically to nails exist, they are usually focused only on the nail plate and are most often applied in conditions such as onychomycosis [44,47,48,55,67,68,69,70,71,72,73,74]. Furthermore, no questionnaire has been developed specifically for ingrown toenails. For this reason, the DLQI can be considered a universal and appropriate tool, allowing for comparisons between patients suffering from different, mainly dermatological, conditions and between various treatment methods for ingrown toenails, whether conservative (applied by dermatologists or podiatrists) or surgical [2,12,38,63]. In conclusion, although the DLQI was not designed specifically for ingrown toenails, it remains the most appropriate available tool, as it captures both dermatological and functional aspects relevant to this condition. Its use also allows for reliable comparison with studies on other nail and skin diseases, ensuring broader scientific validity of the results.

A certain limitation of using the DLQI and other quality-of-life questionnaires is that patients with severe disease may not necessarily perceive a substantial deterioration in their quality of life, for example due to a less demanding lifestyle. Conversely, patients with relatively mild clinical symptoms may experience even small changes as a significant burden. This phenomenon has also been confirmed in the literature, which reports similar findings with the DLQI and other instruments assessing quality of life [13,14,75].

Admittedly, other quality-of-life questionnaires exist that are dedicated to patients with dermatological conditions not limited to the feet and toenails, and these could also have been used in the study [76,77]. However, one of the main advantages of the DLQI is that it adequately captures many dimensions of quality of life [75,78]. In addition, the DLQI is a simple, patient-friendly instrument that is both quick to complete and easy to understand [40]. Importantly, it reflects patients’ subjective experiences and, in line with the intention of its authors, it can serve as a straightforward and rapid tool supplementing the medical interview in everyday clinical practice. It is also designed to become part of a patient’s medical documentation and may be used retrospectively [35,37]. In the present study, patients were often unaware that they were completing an additional, non-standard document, as the DLQI questionnaires were integrated into their medical records. The primary aim of incorporating them was to enrich the clinical documentation with information about patients’ quality of life.

The MCID, discussed earlier in the results section, is also a strong argument for using the DLQI. Unlike many other quality-of-life questionnaires, the DLQI specifies the exact threshold of change in score required to confirm a clinically significant improvement between pre- and post-treatment assessments [40,79]. Thirteen patients in the study did not meet the MCID threshold, which was likely due to their low baseline DLQI1 scores, leaving insufficient room for measurable improvement (a floor effect), rather than indicating a lack of treatment effectiveness. However, in the context of ingrown toenails, achieving the MCID does not determine which treatment approach, conservative or surgical, or which specific surgical technique should be preferred, as no consensus currently exists on this issue [1,80]. It is also worth noting that the authors of other quality-of-life questionnaires frequently use the DLQI as a routine tool in clinical trials, serving as a benchmark for validating or comparing newer instruments [35,38,39,40]. Finally, the availability of a Polish language version of the DLQI was of particular importance for the present study, as it ensured accessibility and comprehensibility for the participating Polish patients [37].

## 5. Conclusions

Plastic surgery of the nail folds preceded by conservative nail plate reconstruction significantly improved quality of life in patients with ingrown toenails, with effects observed across all DLQI domains. The MCID was achieved by most of the patients. Younger age and male gender were associated with better outcomes. The results of the study confirmed that the main domain of quality of life in women suffering from ingrown toenail was embarrassment with their skin and the need to change the way of dressing, while in men, the problem was with practicing sports.

## Figures and Tables

**Table 1:** Characteristics of patients included in the study.

Characteristic	*n* (%)
Gender	
• Female	38 (35%)
• Male	70 (65%)
Number of treated toes	
• One hallux	46 (42.6%)
• Two halluces	62 (57.4%)
Symptom severity (Heifetz classification)	
• Stage 1	5 (4.6%)
• Stage 2	47 (43.6%)
• Stage 3	56 (51.8%)
Duration of symptoms	
• <1 year	20 (18.5%)
• >1 year	88 (81.5%)
Previous surgical treatment for ingrown toenail	
• no surgery	63 (58%)
• surgery	45 (42%)
Presumed cause of ingrown toenail	
• Trauma	11 (10.2%)
• Sports activity	8 (7.4%)
• Incorrect nail cutting technique	42 (38.9%)
• Unknown	42 (38.9%)
• Other	5 (4.6%)

**Table 2:** Structure of the DLQI: Question Domains and Scoring System [35].

Section	Questions	Score
Symptoms and feelings	1–2	Max 6
Daily activities	3–4	Max 6
Leisure	5–6	Max 6
Work and school	7	Max 3
Personal relationships	8–9	Max 6
Treatment	10	Max 3

**Table 3:** Interpretation of the DLQI Scores [35].

Score	Impact on the Patient’s Life
0–1	no impact on patient’s life
2–5	small impact on patient’s life
6–10	moderate impact on patient’s life
11–20	very large impact on patient’s life
21–30	extremely large impact on patient’s life

**Table 4:** No minimal clinically important difference observed between DLQI1 and DLQI2.

Patient Code	DLQI1 Score	DLQI2 Score	DLQI1–DLQI2 Difference
P469	1.0	2.0	−1.0
P482	4.0	3.0	1.0
P493	2.0	0.0	2.0
P504	2.0	0.0	2.0
P508	1.0	0.0	1.0
P513	3.0	1.0	2.0
P514	1.0	0.0	1.0
P515	3.0	0.0	3.0
P540	4.0	2.0	2.0
P561	3.0	0.0	3.0
P563	3.0	1.0	2.0
P578	3.0	0.0	3.0
P579	0.0	0.0	0.0

## Data Availability

The data presented in this study are available on request from the corresponding author due to private medical records of the patients participating in the study. The raw data supporting the conclusions of this article will be made available by the authors on request.
